# Anlotinib for the treatment of recurrent and refractory hemangioblastomas: a case report and review of literature

**DOI:** 10.3389/fonc.2025.1508226

**Published:** 2025-03-18

**Authors:** Qing Tian, Chaofeng Zhou, Shifan Zhou, Sai Wang, Baorong Feng, Keke Zhang, Yu Zhang, Jieqiong Gao, Xiaoyan Mu, Zhizhong Guo

**Affiliations:** ^1^ Henan Province Hospital of Traditional Chinese Medicine (The Second Affiliated Hospital of Henan University of Traditional Chinese Medicine), Zhengzhou, Henan, China; ^2^ Longhua Hospital Shanghai University of Traditional Chinese Medicine, Shanghai, China

**Keywords:** anlotinib, hemangioblastoma, anti-angiogenesis, literature review, case report

## Abstract

Hemangioblastomas (HBs) are rare, highly vascular tumors that present significant treatment challenges, especially in cases of multiple recurrences. Here we present our experience treating one patient with recurrent and refractory HBs, aiming to provide preliminary data on the use of anlotinib in treating this disease. An 18-year-old woman with recurrent and refractory HBs, treated at the Henan Provincial Hospital of Traditional Chinese Medicine, achieved disease remission through oral anlotinib treatment after recurrence. After being diagnosed with HBs, the patient underwent multiple surgical treatments with some efficacy. After recurrence, additional surgery and radiotherapy were not feasible due to the presence of multiple tumor sites. Oral anlotinib (10mg, qd, q3w) resulted in disease remission and significantly improved the patient’s quality of life. Anlotinib may be employed in the management of recurrent and refractory HBs.

## 1 Introduction

Hemangioblastomas (HBs) are tumors originating from mesodermal cells or hemangioblasts (1). HBs are benign vascular tumors that appear sporadically throughout the central nervous system (CNS) or are associated with von Hippel-Lindau (VHL) disease (2). HBs are rare, slow-growing CNS tumors that commonly arise in the cerebellum, brainstem, or spinal cord and less commonly in the spinal canal (3). Surgical resection is the primary treatment for HBs, while stereotactic radiosurgery (SRS) is utilized for surgically unresectable patients (4). For patients with multiple lesions who cannot undergo surgery, systemic therapy may be a viable option. Christian Riklin et al. reported that treatment with the antiangiogenic inhibitor bevacizumab for multiple HBs yielded excellent results after standard treatment failure (5). In this report, we have analyzed the diagnosis and treatment process of a patient with recurrent and refractory HBs treated with oral anlotinib treatment at Henan Provincial Hospital of Traditional Chinese Medicine in 2023, along with a literature review to enhance the understanding of HBs treatment.

## 2 Case presentation

An 18-year-old woman received two craniotomies for microsurgical resection of a space-occupying lesion at Tongji Hospital, Tongji Medical College, Huazhong University of Science and Technology, first in July 2014 and again in April 2022. Histology confirmed the diagnosis of intracranial HB. In July 2022, a follow-up magnetic resonance imaging (MRI) revealed a spinal canal space-occupying lesion in the cervical spine, and the patient subsequently received microsurgical resection of spinal canal space-occupying lesions under general anesthesia at the same hospital. Histology confirmed the diagnosis of spinal canal HBs. Specifically, Immunohistochemistry (IHC) of the April 2022 intracranial HBs showed partial positivity expression for α-inhibin+, VIM+, S-100+, CD56+, and endothelial positivity for CD31 and CD34. It was negative for ERG-, FLT1-, NSE-, Syn-, CgA-, GFAP-, NeuN-, Olig-2-, EMA-, CD10-, PAX-8-, and D2-40-. The Ki-67 labeling index (LI) was approximately 1%. Similarly, IHC of the July 2022 cervical spinal canal HBs showed endothelial positivity for CD31+, CD34+, ERG+, and stromal positivity for α-inhibin+ and S100+. It was negative for D2-40-, CD10-, PAX-8-, Syn-, CgA-, GFAP-, Olig-2-, and EMA-. P53 was scattered positive, and the Ki-67 LI was approximately 1-2%. These findings further support a diagnosis consistent with HBs.

**Figure 1:**
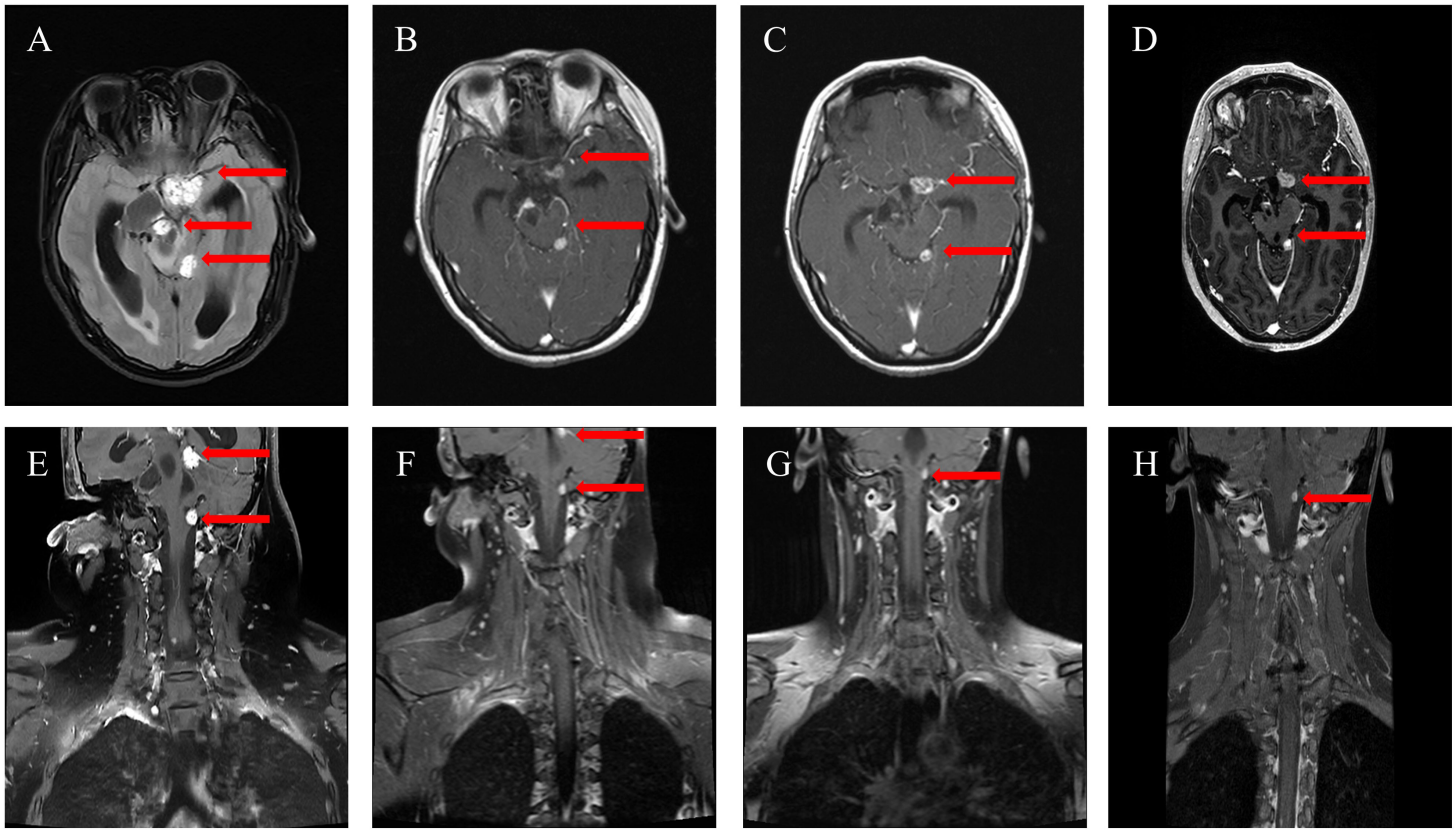
Changes of intracranial and spinal canal HBs of the MRI. **(A, E)** The lesions (red arrow) were observed in October 2023 before anlotinib treatment; **(B, F)** The MRI demonstrated tumor regression (red arrow) after three months of anlotinib in January 2024; **(C, G)** The MRI showed further tumor regression (red arrow) after seven months of anlotinib in May 2024; **(D, H)** The MRI showed continued stability (red arrow) after ten months of anlotinib on August 2024.

Before her presentation, the patient had experienced a gradual onset of subtle symptoms, including narrowing of the palpebral fissures, increased interpupillary distance, and a blunted facial expression, beginning in early January 2023. Subsequently, at the presentation in October 2023, a follow-up MRI revealed multiple abnormal enhancing lesions in the intracranial and spinal canal regions, consistent with tumor recurrence, and demonstrated communicating hydrocephalus, characterized by enlarged lateral ventricles and compression of the cerebral parenchyma (**Figures 1A, E**). Interestingly, the patient did not exhibit typical symptoms of hydrocephalus, such as headache, nausea, vomiting, urinary incontinence, gait instability, or memory impairment. A neurosurgical consultation recommended surgical intervention if necessary. However, given the patient’s history of three prior significant surgeries, her reluctance to undergo further surgery for hydrocephalus, the suspicion that the hydrocephalus was secondary to tumor compression, and the absence of other critical clinical symptoms beyond blurred vision, a decision was made, in agreement with the patient and her family, to pursue medical management aimed at controlling the tumor rather than surgical intervention for the hydrocephalus.

Given that the patient’s neurological examination was notable only for blurred vision and no focal neurological deficits, treatment with oral anlotinib hydrochloride capsules (10mg, qd, q3w) was initiated. A follow-up examination indicated a significant decrease in the MRI’s tumor size after three months (**Figures 1B, F**). A repeat MRI in May 2024 showed further remission, reducing intracranial lesions from three to two and spinal canal lesions from two to one (**Figures 1C, G**). In August 2024, the MRI demonstrated continued condition stability and marked improvement in blurred vision (**Figures 1D, H**). Additionally, the patient exhibited features of trisomy 21 due to hydrocephalus caused by tumor compression and has now returned to a normal appearance.

## 3 Discussion

Intracranial hemangioblastomas (HBs) can develop at any age but are most commonly observed in young and middle-aged individuals, with a peak incidence between 35 and 45 years. The male-to-female incidence ratio is 2:1, with the most common locations being the cerebellar hemispheres, cerebellar vermis, medulla oblongata, and cerebellopontine angle, while supratentorial occurrences are relatively rare. Most HBs are single lesions, with multiple lesions more commonly seen in VHL syndrome, and HBs originating in the spinal cord also often present as multiple lesions (6). The challenge of treating recurrent refractory HBs stems from its complex biological underpinnings. A key factor is the immunosuppressive tumor microenvironment, characterized by the co-expression of PD-1 on infiltrating T cells and PD-L1 on tumor cells, which collaboratively activate immune checkpoint pathways and impair adequate immune clearance of HBs (7). Furthermore, the heterogeneity within HBs tumors, particularly the presence of a CD133+ subpopulation with stem cell-like properties, significantly contributes to recurrence (8). These cells possess self-renewal and differentiation capabilities, resisting conventional therapies and readily leading to HBs tumor relapse (9). Moreover, HBs is a highly vascularized tumor, with its growth and metastasis critically dependent on the formation of abnormal blood vessels (10). The VEGF secreted by HBs cells drives tumor angiogenesis, resulting in structurally disordered and highly permeable ships that provide nutrients for tumor growth and impede the efficient delivery of therapeutic agents within HBs tumors (11). Adding to this complexity, mutations in the VHL gene and the activation of the HIF-1α pathway play a crucial role in the development and progression of sporadic HBs, leading to the overexpression of pro-angiogenic factors like VEGF, which further exacerbates HBs tumor angiogenesis and growth (12).

Despite surgical resection and radiotherapy being the primary treatment modalities, complete removal is often challenging. For solid HBs, particularly those with larger tumors, preoperative tumor-feeding artery embolization may be performed. Vazquez-Ano et al. reported two cases of preoperative embolization for solid HBs at the dorsum of the corticomedullary junction (13). The results indicated that preoperative embolization not only reduced the surgical time and significantly decreased intraoperative tumor bleeding but also facilitated complete tumor resection, minimized damage to adjacent typical structures, and lowered both surgical complications and mortality. For cystic HBs, gross total resection of the tumor nodules is required, and surgical resection is often straightforward. However, it is crucial to meticulously review imaging studies preoperatively and perform thorough intraoperative exploration to avoid missing any nodules when they are small or multiple, which could result in postoperative recurrence. Although HBs are benign tumors, their unique locations and potential for multifocality lead to a relatively high incidence of postoperative complications and mortality.

Clinically, radiotherapy is also employed in treating HBs (14). It is primarily indicated for cases of postoperative tumor residual or recurrence, incompletely resected tumors, tumors located in critical areas such as the brainstem, and patients with multiple tumor sites or significant surgical risks who cannot tolerate surgery. Currently, Gamma Knife radiosurgery and intensity-modulated radiation therapy (IMRT) are commonly used for treating HBs (15, 16). Since HBs are moderately sensitive to radiation, the impact of radiotherapy on the long-term prognosis of HBs patients remains to be studied.

HBs known for their high vascularity and VEGF expression, are potentially responsive to anti-angiogenic therapies (17). Clinical studies have shown the efficacy of agents like pazopanib and intravenous bevacizumab in treating advanced HBs, including VHL-associated and recurrent cases, demonstrating tumor reduction, edema reduction, and improved survival (18–22). Given the established benefits of anti-angiogenic approaches in HBs, anlotinib’s therapeutic efficacy in recurrent refractory HBs can be attributed to its multi-targeted inhibition, which effectively addresses HB-specific mechanisms. As a multi-targeted tyrosine kinase inhibitor, anlotinib potently inhibits VEGFR and blocks other kinases such as PDGFR and FGFR, suppressing HBs tumor angiogenesis through multiple pathways (23). This inhibition of HB tumor vasculature reduces blood supply and improves the tumor microenvironment, enhancing its sensitivity to other therapies. Additionally, anlotinib’s multi-targeted inhibition may affect the self-renewal and differentiation of HBs tumor stem cells, thus hindering HB tumor recurrence (24, 25). While anlotinib does not directly target CD133+ cells within HB tumors, its intervention in multiple signaling pathways may inhibit these stem cell-like cells (26). Concurrently, by inhibiting VEGFR, anlotinib may indirectly regulate the HIF-1α pathway in HBs tumors, reducing VEGF expression and further suppressing angiogenesis (27). Although not a direct immune checkpoint inhibitor, the anti-angiogenic effect of anlotinib may still enhance the anti-tumor activity of immune cells against HBs tumors by modulating the tumor microenvironment and reducing the expression of immunosuppressive factors (28). In summary, anlotinib’s synergistic and multi-faceted mechanisms offer a promising approach to overcome the recurrence and refractoriness of HBs, ultimately leading to therapeutic benefits.

In this case, our patient presented with recurrent and refractory HBs following multiple surgeries. Given the presence of multiple tumor sites, a history of three prior surgeries, and the limited efficacy of radiation therapy, further surgery was deemed high-risk. Moreover, as a high school student, the patient required a convenient outpatient treatment option, making intravenous bevacizumab less suitable. Therefore, to control tumor compression and alleviate vision impairment secondary to hydrocephalus, we initiated oral anlotinib hydrochloride. This decision was based on the patient’s weight of 51 kg, a Karnofsky Performance Status (KPS) score of 80, indicating good functional capacity, and the availability of anlotinib hydrochloride capsules in 12mg, 10mg, and 8mg dosages. Oral anlotinib hydrochloride was started at 10mg once daily, with dose adjustments planned based on tolerability. The patient tolerated the 10mg dose well, and a follow-up MRI after three months revealed significant regression of both intracranial and spinal canal lesions. Specifically, the most extensive intracranial lesion decreased from approximately 1.5 cm to 0.8 cm, and this tumor reduction was accompanied by improved blurred vision.

Furthermore, no adverse effects commonly associated with anti-angiogenic agents were observed. Subsequent imaging in May 2024 demonstrated remission, with the number of intracranial lesions decreasing from three to two and spinal canal lesions from two to one. The most recent follow-up, conducted in August 2024, showed the patient’s condition remained stable, with a notable further improvement in vision. This clinical progress is particularly striking given the patient’s previous presentation with features mimicking trisomy 21 due to hydrocephalus secondary to tumor compression; the patient now exhibits a normal phenotype. This overall improvement is reflected in the patient’s 11 months of enhanced quality of life since tumor recurrence, coupled with a favorable prognosis for continued long-term survival. Consequently, the patient’s survival, quality of life, and psychological well-being have all significantly improved.

Given this, systemic antiangiogenic targeted therapy can still extend survival and improve the quality of life for patients with recurrent and refractory HBs in the spinal canal and brain, where complete surgical resection and radiotherapy are not feasible. While this case study has inherent limitations due to its single-patient nature, it provides valuable clinical insights into the potential efficacy of anlotinib in treating HBs, particularly in cases where conventional therapies are not viable. These observations warrant further investigation through larger-scale clinical trials to validate these findings.

## 4 Conclusion

Only one case report was found in a Medline search (keywords: anlotinib; hemangioblastoma) that showed HBs may respond favorably to oral anlotinib (29). Here, we provided another new report demonstrating that anlotinib results in a positive radiographic response as well as marked clinical improvement when used to treat recurrent and refractory HBs. While the underlying mechanism remains poorly understood, we believe this case report will provide an indication for future research.

## Data Availability

The original contributions presented in the study are included in the article/supplementary material. Further inquiries can be directed to the corresponding authors.
